# Decreased Numbers of Blood Dendritic Cells and Defective Function of Regulatory T Cells in Antineutrophil Cytoplasmic Antibody-Associated Vasculitis

**DOI:** 10.1371/journal.pone.0018734

**Published:** 2011-04-08

**Authors:** Marie Rimbert, Mohamed Hamidou, Cécile Braudeau, Xavier Puéchal, Luis Teixeira, Hélène Caillon, Antoine Néel, Marie Audrain, Loic Guillevin, Régis Josien

**Affiliations:** 1 CHU Nantes, Laboratoire d'Immunologie, Nantes, France; 2 Institut de Transplantation-Urologie-Néphrologie (ITUN), Nantes, France; 3 INSERM, UMR643, Nantes, France; 4 Université de Nantes, Faculté de Médecine, Nantes, France; 5 CHU Nantes, Service de Médecine Interne, Nantes, France; 6 CH Le Mans, Service de Rhumatologie, Le Mans, France; 7 AP-HP, Hôpital Cochin, Service de Médecine Interne, Paris, France; 8 Université Paris Descartes, Paris, France; Pavillon Kirmisson, France

## Abstract

**Background:**

Dendritic cells (DC) and regulatory cells (Treg) play pivotal roles in controlling both normal and autoimmune adaptive immune responses. DC are the main antigen-presenting cells to T cells, and they also control Treg functions. In this study, we examined the frequency and phenotype of DC subsets, and the frequency and function of Treg from patients with ANCA-associated vasculitis (AAV).

**Methodology/Principal Findings:**

Blood samples from 19 untreated patients with AAV during flares and before any immunosuppressive treatment were analyzed, along with 15 AAV patients in remission and 18 age-matched healthy controls. DC and Treg numbers, and phenotypes were assessed by flow cytometry, and in vitro suppressive function of Treg was determined by co-culture assay. When compared to healthy volunteers, absolute numbers of conventional and plasmacytoid DC were decreased in AAV patients. During the acute phase this decrease was significantly more pronounced and was associated with an increased DC expression of CD62L. Absolute numbers of Treg (CD4^+^CD25^high^CD127^low/−^ Tcells) were moderately decreased in patients. FOXP3 and CD39 were expressed at similar levels on Treg from patients as compared to controls. The suppressive function of Treg from AAV patients was dramatically decreased as compared to controls, and this defect was more pronounced during flares than remission. This Treg functional deficiency occurred in the absence of obvious Th17 deviation.

**Conclusion:**

In conclusion, these data show that AAV flares are associated with both a decrease number and altered phenotype of circulating DC and point to a role for Treg functional deficiency in the pathogenesis of AAV.

## Introduction

Antineutrophil cytoplasmic antibodies (ANCA)-associated vasculitides (AAV) are the most common primary systemic small-vessel vasculitis in adults. AAV includes microscopic polyangiitis (MPA), Wegener's granulomatosis (WG), and Churg-Strauss syndrome (CSS). ANCA are directed against constituents of primary granules from neutrophils and against monocytic lysosomes. These can be directed against proteinase 3 (PR3) [1], and against myeloperoxidase (MPO) [2]. PR3 ANCA are mostly found in patients with WG, whereas ANCA with specificity for MPO are associated with MPA and CSS. It is now well accepted that ANCA are directly involved in AAV pathogenesis. ANCA correlate with disease activity [3], and their pathogenic role has been demonstrated in vitro and in vivo [4], [5]. More recently, ANCA with specificity to lysosomal associated membrane protein 2 (LAMP2) were described; interestingly these ANCA appeared to cross react with fimbriated bacteria suggesting a molecular mimicry mechanism [6].

The involvement of T-cells in AAV has also been suggested by several studies [7], [8], [9]. It is well known that ANCA have a T-dependent isotype [10]. Reduced numbers of blood CD4^+^ T cells were observed in AAV, together with a skew toward memory population, which could be the consequence of a persistent activation due to recurrent exposure to an unknown antigen [11], [12]. Recently Abdulahad *et al.* reported a functional defect in CD4^+^ Regulatory T cells (Treg) in patients with Wegener's granulomatosis during remission [13]. So called naturally occurring Treg (CD4^+^CD25^+^FOXP3^+^ T cells) are essential for maintaining peripheral tolerance, preventing autoimmune diseases and limiting chronic inflammatory diseases. These cells suppress the activation and expansion of self-reactive T cells [14], and reduced functional activity of Treg results in an increased susceptibility to autoimmune disease. As the percentage of Treg in patient's peripheral blood can be unaltered when compared with healthy controls, it has been suggested that it is mainly the defective Treg function, rather than their numbers, that contributes to disease development [15]. Patients with multiple sclerosis, polyglandular syndrome type II, active rheumatoid arthritis and type-I diabetes show a significant decrease in the suppressive function of their Treg as compared with cells from healthy donors [15], [16], [17]. In addition, in some autoimmune diseases, reduced numbers of Treg have been observed in the peripheral blood of patients. However, in these cases, the recruitment or migration of Treg from the blood to the inflammatory site may be responsible for the decreased number of Treg in peripheral blood [18]. Thus, quantitative or qualitative defects of Treg may be observed in human autoimmune diseases. Moreover, recent studies suggest that a CD39^+^ subset of Treg could be involved in the control of autoimmune disease-mediated inflammation [19], [20]. Fletcher *et al* reported that these CD39^+^FOXP3^+^ Treg suppressed IL-17 production and were impaired in multiple sclerosis [21].

Dendritic cells (DC) are the key antigen-presenting cells controlling the initiation of the T cell response [22]. DC are not only critical for the induction of primary immune responses, but may also be important for the induction of immunological tolerance, as well as for the regulation of the type of T cell-mediated immune response [23]. In the blood, DC are usually divided into two main populations: conventional DC (also know as myeloid DC, mDC), which are actually heterogeneous, and plasmacytoid DC (pDC) [24], [25]. Whereas mDC produce large amounts of IL-12 and induce Th1 and cytotoxic responses, pDC have a strong capacity for secreting type I interferon in response to viruses [26]. pDC also express the IL-3 receptor αchain (CD123) which is necessary for their survival and differentiation. Involvement of specific receptors on DC in response to particular self-antigens may contribute to the development of an autoimmune vasculitis. Interestingly, Csernok *et al.* showed that PR3 can induce DC maturation in vitro, and can license them for Th1 response potentially favouring granuloma formation in WG [27].

In the present study we assessed firstly, the quantitative and phenotypic modifications of peripheral blood DC, and secondly the numbers, phenotype and function of Treg from patients with ANCA-associated vasculitis in remission or in acute phase compared with age matched healthy individuals. As DC are pivotal in the control of Treg, we hypothesize that both could have quantitative or functional defect in AAV.

## Results

### Decreased numbers of circulating pDC and mDC during AAV

**Figure 1:**
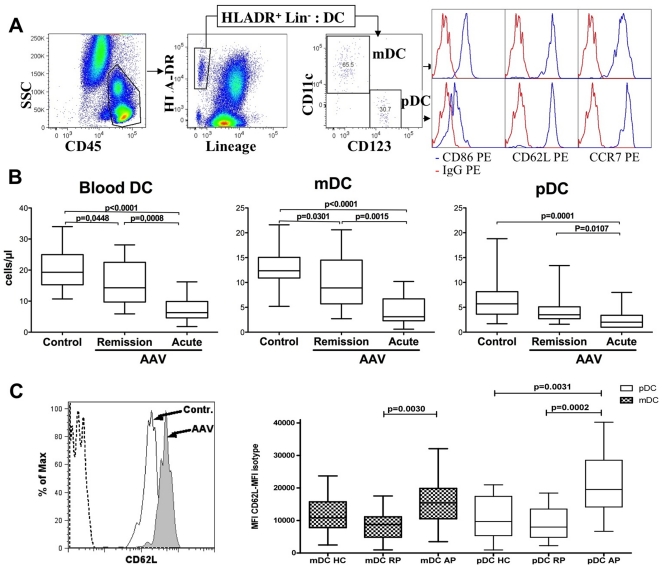
Characterization and count of dendritic cells using flow cytometry. **A**, Gating strategy for identification of DC subsets, and expression of CD86, CD62L and CCR7 (blue line) vs. isotype control (red line) on DC subset. **B**, Quantification of DC, myeloid DC and plasmacytoid DC using flow cytometry is shown for healthy controls (HC ; n = 18), remission phase (RP ; n = 15) and acute phase (AP ; n = 19) AAV patients. Data are presented as boxplots with whiskers from minimum to maximum. **C**, Left: the histogram shows an exemple of CD62 staining on pDC from a control subject (empty histogram) and an AAV patient in acute phase (grey histogram) as compared to isotype control (dotted line); Right: CD62L expression on pDC and mDC is shown as MFI of CDL62 – MFI of isotype control. Data are presented as boxplots with whiskers from minimum to maximum.

Circulating DC are a rare population of blood leukocytes (<1% of PBMC), which can be identified as Lin^−^ HLA-DR^+^ cells. Two subsets of DC can be separated on the basis of CD11c and CD123 expression: so-called myeloid or conventional DC that are CD11c^+^ CD123^−^ and plasmacytoid DC (pDC) that are CD11c^−^ CD123^+^ (**Fig. 1A**). The numbers of circulating DC subsets were assessed on fresh whole blood samples from controls and patients after staining with CD45, Lin, HLA-DR, CD11c and CD123, and using calibrated beads. When compared to healthy controls (HC), AAV-patients exhibited a significant decrease in total DC numbers both during the acute phase (AP) and the remission phase (RP) (7.5 DC/µL *vs* 20 DC/µL, *p*<0.0001 ; 15.5 cells/µL *vs* 20 DC/µL ; *p* = 0.0448). Moreover, the decrease in DC between the 2 groups was significant, with a more pronounced decrease seen during the acute phase than during the remission phase (7.5 DC/µL *vs* 15.5 cells/µL *p* = 0.0008). The same significant decrease was observed for mDC between HC and RP (*p* = 0.0301), between HC and AP (*p*<0.0001), and also between RP and AP (*p* = 0.0015). For pDC the decrease was significant only in AP *versus* HC (*p* = 0.0001), and AP *versus* RP (*p* = 0.0107) (**Fig. 1B**).

### Increased expression of CD62L on DC during acute phase of AAV

To address the activation and the maturation status of circulating DC, we assessed the expression of CD86, CD62L and CCR7 on blood DC. CD86 (B7-2) is an important costimulatory molecule that is upregulated on mature DC. CCR7 is a crucial homing molecule for addressing DC to secondary lymphoid organs and CD62L was shown mediate pDC recruitment to inflamed lymph nodes [28] and in vivo monocyte-derived DC migration to lymph nodes [29].

It is well known that blood DC have a semi mature phenotype characterised by an expression of CD86 and CCR7 but a lack of CD83. There was no significant difference between AAV patients and controls regarding the expression of CD86, and CCR7 on DC. The large majority of mDC, but few pDC, were CD86^+^ (94%, 93%, 90% on mDC, and 28%, 31%, 31% on pDC in HC, RP, AP respectively). The expression levels of CD86 on DC were equivalent for patients and controls. Between 82 and 92% of DC were CCR7^+^ and the expression levels were not significantly different between patients and controls (data not shown). No surface expression of CD83 was detected on DC from patients or controls (data not shown). Although virtually all mDC and pDC expressed CD62L in the three groups of individuals, we observed a significant increase in CD62L expression levels on both pDC and mDC from acute phase patients as compared to patients in remission (*p* = 0.0002 and *p* = 0.0030 respectively). This difference was more pronounced for pDC than for mDC (**Fig. 1C**).

### Decreased numbers but equivalent frequencies of circulating Treg cells during acute phase of AAV

**Figure 2:**
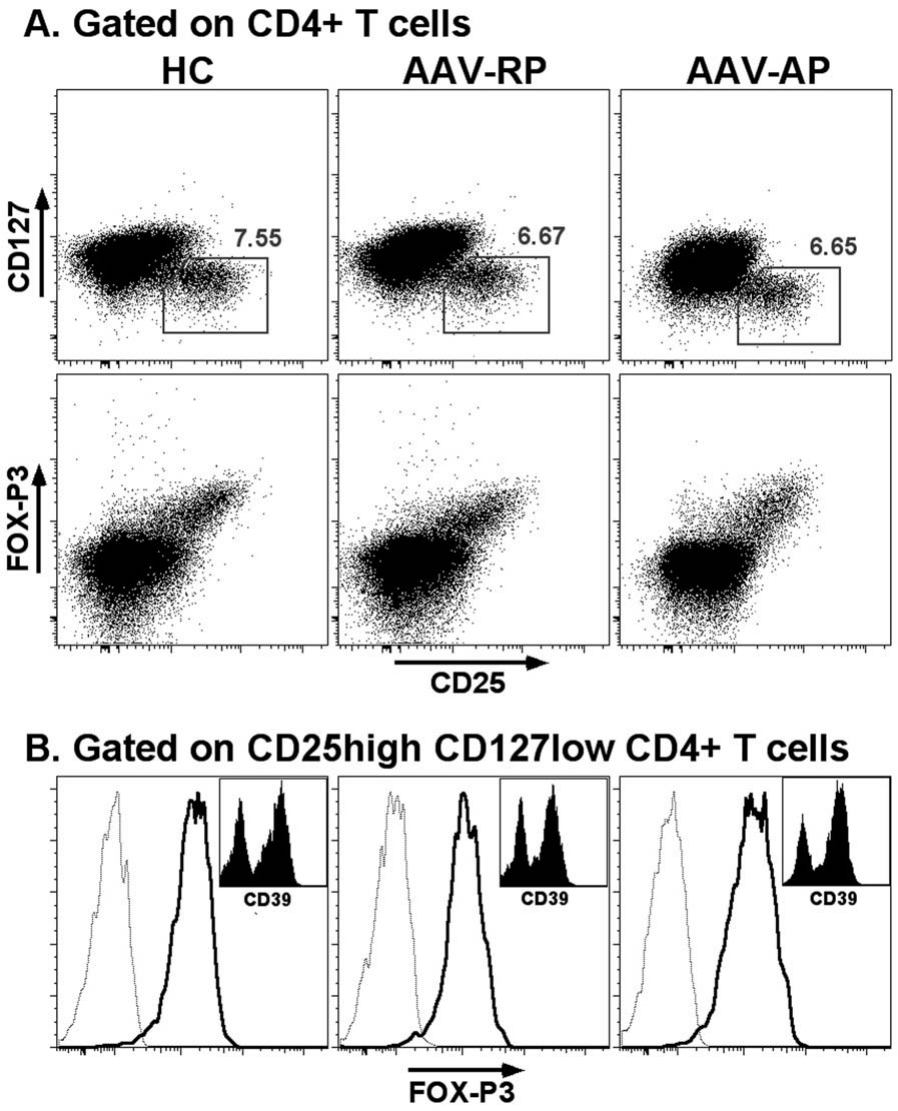
Characterization of Treg using flow cytometry in controls and patients. **A**, Expression of CD25, CD127 and FOX-P3 in CD4^+^ T cells. Representative dot plots for healthy controls (HC), AAV patients in remission phase (AAV-RP) and AAV patients in acute phase (AAV-AP) showing CD25 vs. CD127 and CD25 vs. FOXP3 expression in gated CD4^+^CD3^+^ T cells are shown. The gate use to identify Treg in fresh blood is shown in each CD25 vs. CD127 dot plot. **B**. Representative histograms showing expression of FOX-P3 (bold line) vs. isotype control (dotted line) and CD39 (inset) in CD4^+^CD25^high^CD127 ^low^ cells as gated in A.

**Figure 3:**
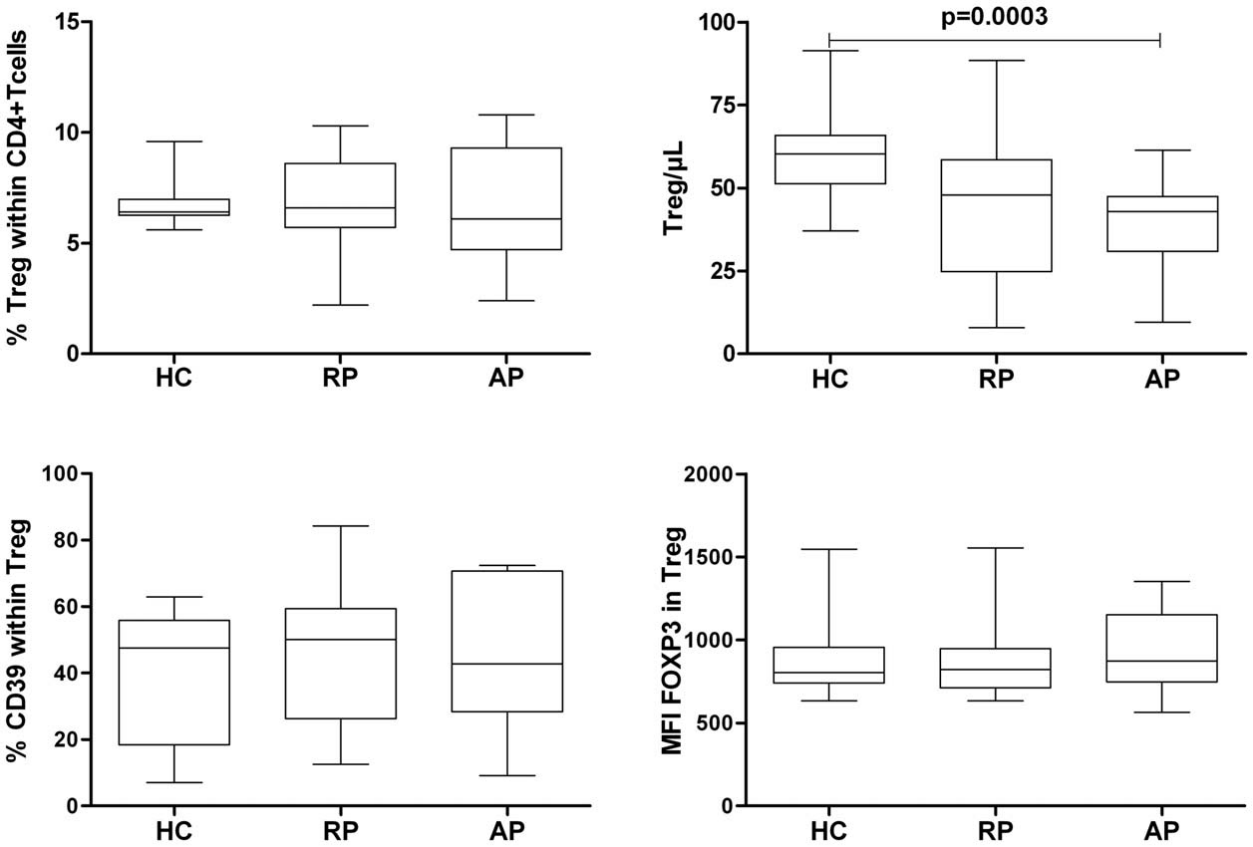
Frequency, number and phenotype of Treg in patients vs. controls. Histograms represent: **A**, Proportion of CD4^+^CD25^high^CD127^low/−^ T cells within CD4^+^CD3^+^ T cells (as gated in figure 2A) for HC (mean, 6.8%, *n* = 18), remission, (mean, 6.9%, *n* = 15), and acute (mean, 6.7%, *n* = 19) AAV patients. No significant difference was observed for percentage of Treg cells between the 3 groups. **B**, Absolute numbers of CD4^+^CD25^high^CD127^low/−^ T cells in whole blood for HC (mean, 60.3 cells/µL), remission (mean, 47.5 cells/µL), and acute (mean, 40.1cells/µL) AAV patients. **C**, MFI of FOX-P3 in gated Treg and **D**, Mean percentage of CD39^+^ cells within Treg. Data are presented as boxplots with whiskers from minimum to maximum.

The frequencies and the absolute numbers of circulating Treg were assessed in whole blood samples. Tregs were identified among CD4^+^CD3^+^ cells as CD25^high^CD127^low/−^ cells. The gating strategy use in controls and AAV patients is shown in figure 2. Previous studies have shown that CD25 expression was upregulated on CD4^+^ T cells from AAV patients [12], [30] as in other inflammatory disease. This was indeed the case in our study with CD4^+^ T cells from AP patients. In contrast, CD25/CD127 profiles for remission patients were similar to that observed in controls (**Fig. 2A**). Therefore, in most of acute phase patients, a slight modification of the Treg gate was necessary to account for this increase in CD25 expression (**Fig. 2A**). We confirmed using PBMC that these gated cells expressed similar and high levels of FOXP3 in controls and in AAV-patients (**Fig. 2B**). Although the frequencies of Treg among CD4^+^ T cells were not significantly different between controls and patients (**Fig. 3A**), a significant decrease in absolute numbers of Treg was observed in patients during acute phase as compared to control individuals (**Fig. 3.B**; *p* =  0.0003). However, this decrease was correlated with the lymphopenia usually observed in these patients. The frequencies and levels of FOX-P3 expression in CD3^+^CD4^+^CD25^high^CD127^low/−^ cells were compared on PBMC and we did not observed any differences between controls and AAV patients (n = 10 for each group) (**Fig. 3C**). We also assessed the expression of CD39 in CD3^+^CD4^+^CD25^high^CD127^low/−^ Treg cells on cryopreserved PBMC. Again, no differences were observed between the 3 groups with 40%, 45% and 42% of Treg expressing CD39 in controls, acute phase AAV and remission AAV, respectively (**Fig. 3D**).

### Defective suppressive function of circulating Treg cells in AAV-patients in remission and in acute phase

**Figure 4:**
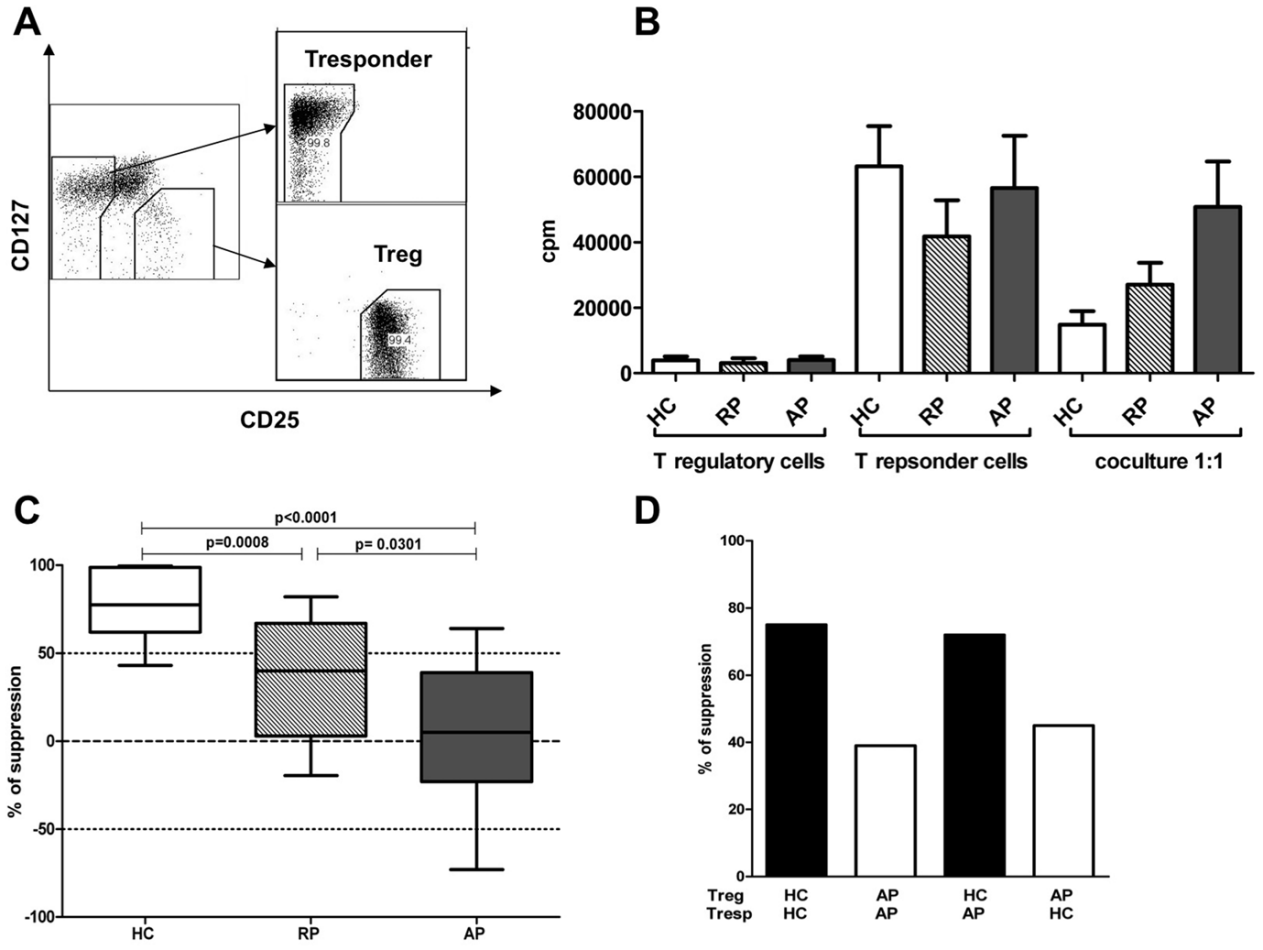
Suppressive function of circulating T regulatory cells. **A**, Gating strategy for Treg sorting and typical results after sorting. **B**, Proliferative responses of Treg, effector responder T cells and Treg:Tresp co-cultures (ratio1/1) upon CD3+CD28 stimulation. Histograms are expressed as mean value +/− SD of cpm for healthy controls (HC ; n = 18), remission patients (RP ; n = 15), and active patients (PA ; n = 19). **C**, The percentage of suppression of responding cells was determined as 1−(proliferation of co-culture/proliferation of responder population alone)×100. Data are presented as boxplots with whiskers from minimum to maximum. **D**, A suppressive assay mixing Treg and effector T cells from and AP patient and a healthy volunteer was performed. Black bars represent suppression with HC Treg, and white bars with patient Treg. The results of one experiment out of three with similar results are shown.

We then addressed the in vitro suppressive activity of Treg using a polyclonal stimulation assay. Treg were isolated by FACS as CD4^+^CD25^high^ and CD127^low/−^ cells (**Fig. 4A**). T cells were stimulated by anti-CD3 in the presence of soluble anti-CD28 mAb. Treg alone incorporated less than 5,000 cpm. Means of proliferation for T responder cells alone were 63,200 for healthy donors, 41,760 for remission patients and 56,600 for acute phase patients. For co-culture assays, the proliferation was higher for patients in acute phase and in remission (respectively 50,769 and 27,020) than in controls (14,808) (**Fig. 4B**). As expected, Treg from controls exhibited a potent suppressive activity in vitro when mixed at 1∶1 ratio with effector T cells. In contrast, Treg from patients exhibited a decrease in their suppressive activity. Acute phase patients presented a significant decrease in suppressive activity as compared to both controls (7% *vs* 78%; *p*<0.0001) and the remission group (7% *vs* 37%; p = 0.0301). In addition, the remission group exhibited a decreased Treg suppressive function as compared to controls (37% *vs* 78%. *p* = 0.0008) (**Fig. 4C**). To determine whether the observed defect in suppression was related to patient Treg intrinsic defective suppression or to a resistance of patient effector cells to Treg-mediated suppression, we performed suppressive assays with Treg from patient and effector cells from control and *vice versa*. At a ratio of 1∶1 HC Treg suppressed patient effector cells by 72% (compared with 75% inhibition of autologous HC effector T cells), whereas patient Treg cells were able to suppress HC effector cell proliferation by only 45% (39% of autologous effector cells). This indicates that the suppressive function of Treg from AAV patients was indeed intrinsically defective (**Fig. 4D**).

### Lack of Th17 deviation in AAV

**Figure 5:**
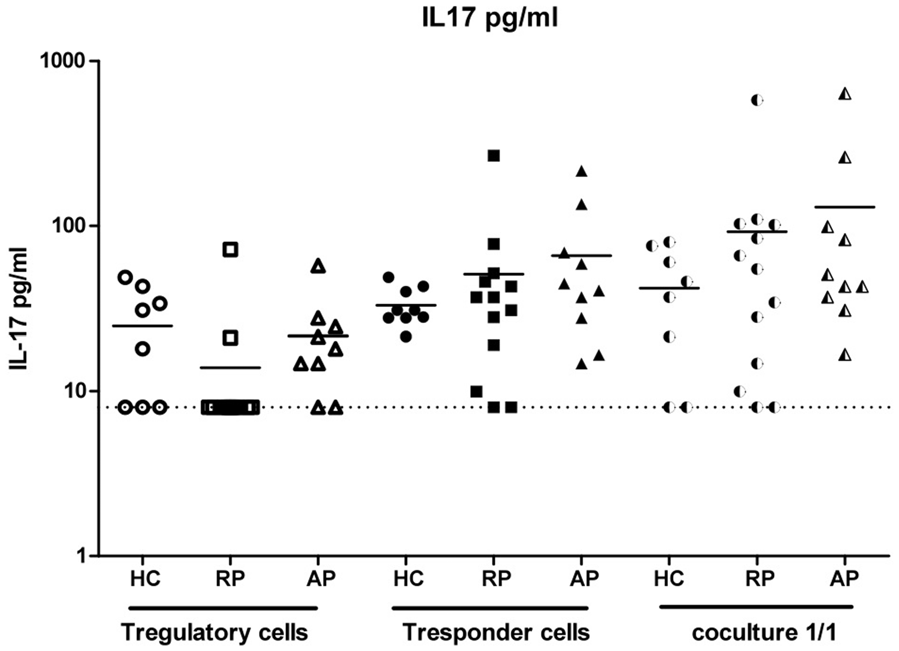
Concentration of IL-17 in co-culture supernatants. IL-17 was quantified in supernatant using a multiplex fluorescent bead immunoassay: the levels of IL-17 produced by Treg, responder T cells and co-culture were not statistically different between the 3 groups.

Finally, we sought to determine whether the defective Treg function observed in AAV patients could be associated with a Th17 deviation. The in vitro production of IL-17 by patients and control CD4^+^ T cells subsets after 4 days of stimulation by anti-CD3+CD28 mAbs was assessed. As shown in **Fig. 5**, the levels of IL-17 produced by CD4^+^CD25^−^ T cells were not statistically different between the 3 groups although there was a tendency to higher IL-17 secretion by T cells from acute phase patients. CD4^+^CD25^high^CD127^low/−^ produced less IL-17 than CD25^−^CD4^+^ T cells, again with no significant difference between the three groups. Finally, co-culture of Treg with effector T cells did not result in the suppression of IL-17 production in any group. We also assessed the intracellular expression of IL-17 in CD4+ T cells by flow cytometry after polyclonal stimulation (PMA + ionomycine) of frozen PBMCs, and again found no differences between controls and patients (data not shown). Therefore, our data suggest that the defect in suppressive function of Treg in AAV patients is not associated with a Th17 deviation.

## Discussion

Dendritic cells and Treg have been shown to play critical roles in the control and pathogenesis of autoimmune diseases. To our knowledge, this is the first report on the frequency and phenotype of circulating DC in AAV. Both mDC and pDC numbers were reduced in AAV patients during acute phase and even, although at a lesser extent, during remission. Our results also confirmed that Treg function is impaired in AAV during remission and more importantly, showed that this function is even more dramatically reduced in acute phase patients that did not received any immunosuppressive drug.

Animal models have shown that DC play a pivotal role in the induction and in the maintenance of peripheral T cell tolerance. Various mechanisms have been demonstrated such as induction of anergy, deletion or regulatory T cells. Moreover, DC were also shown to control the function of naturally induced regulatory T cells (Treg) during immune response. The role of T cells in the induction of AAV has been suggested by many studies [4]. A reduction in circulating DC subset numbers have been observed in various autoimmune diseases such as systemic lupus erythematosus [31], [32], celiac disease [33] rheumatoid arthritis and psoriatic arthritis [34], [35]. Such a decrease in circulating DC could be related to a reduction of DC differentiation from bone marrow progenitors, to a reduced global half-life of DC or to a recruitment of blood DC to either lymphoid or non lymphoid tissues. A significant decrease in DC numbers was observed in AAV patients, both during the acute phase and the remission phase. However, the phenotype of both mDC and pDC from remission patients, which can be defined as semi mature, was not different from that observed in healthy donors. Interestingly, during the acute phase the decrease in the number of DC was more pronounced, and was associated with an increase in CD62L expression. Because CD62L was previously shown to mediate pDC migration to lymph nodes [28], this altered phenotype in AAV patients could favor their recruitment in secondary lymphoid organs. The role of CD62L in circulating mDC migration remains to be established. In contrast to our results, expression of CD62L was reduced on DC from patients with psoriatic arthritis and with rheumatoid arthritis [34], indicating that variation in CD62L expression in AAV patients can not be simply related to inflammatory process. A recent study suggests that immature DC are recruited in the kidneys of AAV patients, and then cluster with T cells in the interstitial infiltrate [36]. Therefore, the reduced DC numbers we observed during the acute phase could be related to their recruitment in secondary lymphoid organs and/or in inflammatory tissues. It is interesting to note that this feature persisted during remission although a role for low doses of corticosteroids in reducing DC numbers is possible. Once in secondary lymphoid organs, it is possible that DC could modulate T-cell responses during AAV. Other mechanisms that could explain this decreased DC numbers, i.e as defective DC differentiation, and decreased DC half-life still need to be explored.

Treg suppress the activation of conventional T cells in vitro and play a key role in vivo in regulating the immune responses that drive autoimmune diseases. Abdulahad *et al* reported that WG patients in remission exhibited an increase in the proportion of memory-like Treg (FOXP3^+^, CD25^high^, CD45RO^+^) among CD4^+^ T cells that are functionally defective [13]. The absolute numbers were not reported in this study, and several patients were treated by immunosuppressive treatments. However, the authors suggested that these FOXP3^+^ CD25^high^ cells could be chronically activated T cells rather than true Treg. In contrast, we observed a moderate decrease in the absolute numbers of circulating Treg in acute phase patients, whereas their proportion among CD4^+^ T cells were not modified when compared to healthy controls. These discrepancies might be related to the fact that we performed our study on whole blood and used the CD25 and CD127 markers to identify Treg, whereas Abdulahad *et al.* used PBMC, with CD25 and FOXP3^+^ as markers. It should be noted that the moderate decrease in Treg numbers in AAV patients was likely due to the global lymphopenia that is usually observed in these patients, especially during flares, whereas the frequency of Tregs within the CD4 compartment was similar to that of controls. We furthermore found that the expression level of FOXP3^+^ in CD3^+^CD4^+^CD25^high^CD127^low/−^ cells was similar in AAV patients and controls. More importantly, the ex vivo suppressive function of Treg from AAV patients was defective. In remission patients without immunosuppressive therapy or with low dose of corticosteroids only, this defect was actually moderate; therefore our results confirm the previous studies that showed a defective Treg function in WG patients and extend this finding to non WG AAV [13], [37], [38]. In addition, this defect was more dramatic during acute phase. Importantly, these patients were analysed before the administration of any immunosuppressive drugs which could obviously strongly modify in vitro assays.

There are controversies regarding the assessment of suppressive function of Treg in autoimmune diseases, which clearly depends on the markers used to sort Treg. For instance, in multiple sclerosis, Treg cells isolated by CD4^+^CD25^high^ expression had impaired function [39], but Treg cells defined as CD4^+^CD25^high^CD127^low/−^ had the same suppressive function compared to controls [40]. In fact, a general increase in CD25 expression in T cells has been reported in various inflammatory diseases including AAV [12], [30]. Therefore, CD25 is not a suitable marker by itself to identify Treg in human [41]. Moreover, FOXP3 may also be transiently upregulated in human activated effector [42]. Treg cells were sorted in our study on the basis of CD25 and CD127 expression, which was shown to help discriminating Treg from effector T cells [43]. However, CD127 could also be down regulated on activated effector T cells [41]. Although we could therefore not completely exclude that sorted Treg might be contaminated by small numbers of effector T cells, it should be noted that CD25 expression was not upregulated in AAV remission patients and that their CD25/CD127 profiles were similar to that observed in controls. Yet, even in these patients, the in vitro suppressive activity of sorted Tregs was strongly reduced as compared to controls. Additional studies are necessary to correlate this impaired suppressive function with a more detailed phenotype of Treg in AAV patients together with potential changes in FOX-P3^+^ Treg subset representation [41].

These results point to a possible role for Treg functional deficiency in the pathogenesis of AAV and relapses. Development of naturally occurring Treg cells occurs in the thymus, and their maintenance and homeostasis possibly depends on the recognition of auto-antigens presented by DC in peripheral lymphoid organs. The suppressive mechanisms by which Treg cells exert their inhibitory effects in vitro were found to be dependent on a direct T-regulatory/T-effector cell contact. The inhibitory effect itself is antigen nonspecific but requires antigen-dependent activation of the regulatory T cells through their T-cell receptor. In this respect, antigen presenting cells play a crucial role in activation of Treg, and previous studies have shown that DC can actually control Treg function [44], [45], [46], [47]. Murine immature DC were shown to induce Treg suppressive activity in vitro whereas following DC maturation induced by TLR ligation, Treg suppressive activity was inhibited [48]. However, mature DC are potent inducers of Treg proliferation, and mature–DC expanded Treg appeared to retain their suppressive activity [46], [49]. This is consistent with the fact that costimulation signals are essential to survival of functional Tregs in vivo [50]. In fact, recent murine data indicate that, in vivo, the numbers of Treg are crucially dependent on the numbers of DC [51]. The recent description of a new syndrome of DC, monocyte and NK lymphoid deficiency in human supported this role of DC in Treg homeostasis [52]. Indeed, in these patients, the lack of DC was associated with a strongly reduced numbers of circulating Treg. Finally, it is possible that conventional and plasmacytoid DC have different roles in regulating Treg homeostasis and functions [53]. How this DC-Treg feedback loop is affected during inflammatory diseases remains to be determined.

Importantly, the defect of suppressive activity in AAV patients was Treg-intrinsic and not related to a relative resistance of effector T cells to Treg-mediated suppression. Several studies have shown that Treg can be separated into subsets based on the expression of markers such as CD103, ICOS or CD39 [20], [54], [55], [56]. It was shown that CD39^+^ but not CD39^−^ Treg were able to suppress Th17 activation, and that CD39^+^ Treg were decreased in multiple sclerosis patients [21]. However, we showed here that this is not the case in AAV patients. Th17 cells, which are a subset of effector CD4+ T cells specialized in the production of IL-17 and IL-22 and in recruitment and activation of neutrophils, have been implicated in autoimmune diseases, including multiple sclerosis, lupus and diabetes [57]. A recent study suggested that T cells from ANCA-positive WG patients also exhibited a skewed Th17 response following stimulation with the auto-antigen PR3 [58]. More recently, Nogueira *et al* reported an increase in the level of IL-17 and IL-23 in the sera of AAV patients [59]. This is a tempting hypothesis as it is well known that Treg and Th17 are developmentally related [60], [61], [62], [63]. We further assessed this Th17/Treg balance hypothesis, by analysing the production of IL-17 by effector T cells from AAV patients following polyclonal stimulation. We could not find any evidence of Th17 deviation, both in remission and in acute phase patients. Therefore the possible role of Th17 in AAV needs to be clarified by further studies.

In conclusion, we demonstrated that DC numbers, as well as Treg suppressive activity, are significantly reduced in untreated patients with AAV in a disease activity specific manner. The decrease in Treg suppressive function might be related to changes in DC function, and might be an important defective tolerance checkpoint in AAV.

## Materials and Methods

### Study population

Blood samples from 19 untreated patients with AAV in acute phase (AP) with a BVAS above 3 (Birmingham Vasculitis Activity Score), 15 AAV patients in remission (RP, BVAS<3) and 18 age-matched healthy controls (HC) were analysed (Table 1). The diagnosis of AAV was established according to the Chapel Hill criteria, and all patients had ANCAs at this time. For patients in acute phase, either at presentation or during relapse, samples were drawn before any immunosuppressive therapy (included corticosteroids). Patients in remission included in this study either did not receive any immunosuppressant (11/15) or received corticosteroids only with a maximum dosage 10 mg/d (4/15).

**Table 1:** Clinical features of patients.

	Patients in remission (BVAS<3)	Patients in acute phase (BVAS>3)	Healthy controls
Mean age (years)	58	57	55
**Wegener's granulomatosis**	**11**	**14**	**-**
ANCA anti-PR3	7	11	-
ANCA anti-MPO	4	3	-
**Microscopic polyangiitis**	**4**	**5**	**-**
ANCA anti-PR3	-	-	-
ANCA anti-MPO	3	5	-
**Patient number**	**15**	**19**	**18**

Ethics statements: all patients and healthy controls provided written informed consent. Internal board and the local medical ethics committee (Comité de Protection des Personnes Ouest IV, Nantes) reviewed and specifically approved the study.

### Dendritic cells and regulatory T cells characterisation

Circulating dendritic cells and regulatory T cell numbers were measured by flow cytometry on fresh whole blood within 24 h after sampling. Whole blood cells were incubated with monoclonal antibodies (mAbs) followed by erythrocyte lysis with FACS lysing solution (BD Biosciences, San Jose, CA, USA) at room temperature. After washing with PBS, events were analysed in a LSRII cytometer (BD Biosciences). Absolute counts of lymphocytes/monocytes and CD4 lymphocytes were determined with BD Multitest™CD3/CD8/CD45/CD4 in BD Trucount™ Tubes.

To identify dendritic cells, a six-color flow cytometry assay was performed with the following mouse anti-human mAbs: fluorescein isothiocyanate (FITC)-conjugated lineage cocktail-1 Abs (Lin 1), Am Cyan conjugated anti-CD45 (clone HI30), Allophycocyanin (APC)-conjugated CD11c (clone S-HCL-3), phycoerythrin-cyanin5 (PECy5)-conjugated anti-CD123 (clone 9F5), Allophycocyanin cyanin7 (APC-Cy7)-conjugated anti-HLA-DR (clone L243) and Phycoerythrin (PE)-conjugated with CD86 (clone 2331), CD62L (clone DREG56), CCR7 (clone 150503) or IgG (1+2a) (all from BD Biosciences, except CCR7, R&D systems, Minneapolis, MN, USA and IgG (1+2a)-PE from Beckman Coulter, Miami FL). The Lin1 contained several mAbs: CD3 (T cells; clone SK7), CD14 (monocytes/macrophages; clone MΦP9), CD16 (natural killer cells; clone 3G8), CD19 (B cells; clone SJ25C1), and CD56 (natural killer cells, clone NCAM16.2). Dendritic Cells were identified as Lin^−^HLA-DR^+^ cells within a lymphocytes-monocytes gate. CD11c and CD123 expression was determined within Lin^−^HLA-DR^+^ cells in order to define myeloid DC (CD11c^+^CD123^−^) and plasmacytoid DC (CD11c^−^CD123^+^) subsets. Absolute DC numbers were calculated from the lymphocyte-monocyte counts. In each subtype, we analysed the percentage and the expression level of CD86, CD62L and CCR7. The expression levels were calculated by subtracting the mean fluorescence intensity (MFI) of the isotype from the MFIs of each marker (**Fig. 1A**).

Treg cells were identified with Peridinin chlorophyl protein (PerCP)-conjugated anti-CD45 (clone 2D1), Phycoerythrin (PE)-conjugated anti-CD127 (clone SB/199), phycoerythrin-cyanin7 (PC7)-conjugated anti CD25 (clone 2A3), and fluorescein isothiocyanate (FITC)-conjugated anti-CD3 (cloneSK7), all from BD Biosciences, and Allophycocyanin-H7 (APC-H7)-conjugated anti-CD4 (clone 13B8.2) from Beckman Coulter. Treg were identified as CD45^+^CD3^+^CD4^+^CD25^high^CD127^low/−^. The number of Treg were deduced from the CD4 T cells number multiplied by the proportion of regulatory T cells in CD4 cells. To assess the expression of FOXP3 and CD39 in Treg, frozen PBMC were labelled with CD4-APC-H7, CD127-FITC, CD25-PC7 (same clone as above) and CD39-APC (clone TU66), all antibodies from BD Biosciences, followed by fixation, permeabilization, and intracellular staining with FOXP3 mAb (clone PCH101, eBiosciences, San Diego, CA) according to the manufacturer's instructions (**Fig. 2**). This last staining on PBMC was performed on 10 Healthy controls, 10 patients with AAV in acute phase, and 10 patients with AAV in remission.

### CD4^+^CD25^−^, CD4^+^CD25^high^CD127^low/−^ Cell sorting

PBMCs were isolated from EDTA whole blood by density centrifugation over Ficoll-Paque (Eurobio). 45×10^6^ freshly isolated PBMC were incubated for 10 min in 100 µL of PBS- EDTA with anti-CD4 FITC (clone RPA-T4 BD biosciences), CD127 PE (clone SB/199 BD biosciences) and anti-CD25 AlexaFluor 647 (in house clone 33B3.1 conjugated to AF647 using a kit from Invitrogen). Human CD4^+^CD25^−^ (Responder T cells) and CD4^+^CD25^high^CD127^low/−^ were then separated using a high-speed cell sorter (FACSAria; BD Biosciences). Purity was routinely >98% for Treg and >99% for CD4+CD25− T cells (**Fig. 3A**).

### In vitro suppression assay

Suppressive function of Treg was determined by co-culture assay, with all experiments performed on fresh peripheral blood lymphocytes. To assess the functional activity of T regulatory cells, 2×10^4^ responder cells were co-cultured for 4 days with 2×10^4^ Treg cells (ratio 1∶1) in complete RPMI 1640 medium supplemented with HEPES, L-glutamine, penicillin, streptomycin, sodium pyruvate, non-essential amino acids, and 10% human AB serum. All assays were performed in anti-CD3 (Orthoclone OKT3; Janssen-Cilag, 10 µg/mL) 96-well round-bottom plates in a final volume of 200 µL of complete medium, with soluble anti-CD28 at 5 µg/ml (clone CD28.2 BD Biosciences). After 4 days of culture, the cells were pulsed with 1 µCi per well of ^3^H-thymidine (Amersham Biosciences) for 8 hours. The cells were then harvested and counted in a scintillation counter. ^3^H-thymidine incorporation was measured as cpm. The percentage of suppression of the responding cell proliferation was determined as 1−(mean cpm of co-culture/mean cpm of responder alone)×100.

### Cytokines

IL-17 was measured in supernatants of co-culture 4 days after the initiation using a multiplex fluorescent bead immunoassay (Biosource) together with a Luminex cytometer. For this experiment, supernatants of 10 HC, 14 RP and 11 AP were analysed.

### Statistical analysis

Statistical comparisons between pairs of experimental groups were performed using the Mann-Whitney U-test for unpaired data, and a *P*-value of less than 0.05 was considered statistically significant. Data are presented as boxplots with whiskers from minimum to maximum.
